# *Trim46* contributes to the midbrain development via Sonic Hedgehog signaling pathway in zebrafish embryos

**DOI:** 10.1080/19768354.2021.1889661

**Published:** 2021-03-01

**Authors:** Jangham Jung, Jaehun Kim, Tae-Lin Huh, Myungchull Rhee

**Affiliations:** a Department of Life Science, BK21 Plus Program, Graduate School, Daejeon, South Korea; b Department of Biological Sciences, College of Bioscience and Biotechnology, Chungnam National University, Daejeon, South Korea; c School of Life Sciences and Biotechnology, College of Natural Sciences, Kyungpook National University, Daegu, South Korea

**Keywords:** *Trim46a*, Sonic Hedgehog (SHH), Foxa2, midbrain, MHB (Midbrain-Hindbrain boundary), cyclopamine

## Abstract

TRIM46 is a RING finger E3 ligase which belongs to TRIM (tripartite motif-containing) protein family. TRIM46 is required for neuronal polarity and axon specification by driving the formation of parallel microtubule arrays, whereas its embryological functions remain to be determined yet. Expression patterns and biological functions of *trim46a*, a zebrafish homologue of TRIM46, were studied in zebrafish embryo. First, maternal transcripts of *trim46a* were present at 1 cell stage whereas zygotic messages were abundant in the eyes, MHB (Midbrain-Hindbrain Boundary) and hindbrain at 24 hpf (hours post fertilization). Second, transcriptional regulatory region of *trim46a* contains *cis*-acting elements binding a transcriptional factor Foxa2. Transcription of *foxa2* is positively regulated by Sonic Hedgehog (SHH), and treatment of cyclopamine, an SHH inhibitor, represses transcription of *foxa2* in 4 hpf through 24 hpf embryos. Third, the transcriptional repression of *foxa2* inhibited transcription of *trim46a* to cause developmental defects in the midbrain and MHB. Finally, spatiotemporal expression patterns of a midbrain marker *otx2b* in the developmental defects confirmed inhibition of SHH by cyclopamine caused underdevelopment of the midbrain and MHB at 24 hpf. We propose a signaling network where *trim46a* contributes to development of the midbrain and MHB via Foxa2, a downstream element of SHH signaling in zebrafish embryogenesis.

## Introduction

Eukaryotic cells are equipped to degrade proteins via the ubiquitin-proteasome system (UPS). Proteins become degraded upon their conjugation to chains of ubiquitin where they are directed to the 26S proteasome, a macromolecular protease (Deshaies and Joazeiro 2009; Kleiger and Mayor 2014). The post-translational attachment of ubiquitin, a highly conserved 76-amino-acid polypeptide, also directs myriad eukaryotic proteins to a variety of fates and functions, such as internalization and lysosomal targeting, modulation of protein interactions, alteration of subcellular distribution, regulation of transcription, DNA repair and propagation of transmembrane signaling, most notably in the nuclear factor kappa B (NF-κB) pathway (Metzger et al. 2012). Derangements of the UPS can lead to the dysregulation of cellular homeostasis and the development of multiple disorders including neurodegenerative and systemic autoimmune diseases (Nalepa et al. 2006).

TRIM46 (tripartite motif-containing 46) belongs to the large family of tripartite motif proteins that have been implicated in many biological processes including UPS, cell differentiation, transcriptional regulation and signaling pathway (McNab et al. 2011; Versteeg et al. 2013). TRIM proteins are characterized by the presence of a highly conserved N-terminal RBCC motif that consists of a RING finger domain, one or two B-boxes, and a coiled-coil domain but differ in their C-terminal regions that confer functional specificity (Reymond et al. 2001; Meroni and Diez-Roux 2005; Short and Cox 2006). Based on their C-terminal domain composition, TRIM46 has been classified into C11 subgroups (Ozato et al. 2008).

Ubiquitin ligase TRIM46 has been revealed as an essential element in neurogenesis as well as in the initiation and maintenance of axonal transport. TRIM46 is localized in the proximal axon, where it specifies neuronal polarity of microtubule bundles oriented with their plus-end pointing outward (Curcio and Bradke 2015), defining an axonal cytoskeletal compartment for microtubule organization during neuronal development (van Beuningen et al. 2015). TRIM46 regulates microtubule fasciculation in the axon initial segment (AIS) (Harterink et al. 2019) and is transported by microtubule-based motors KIF3A/B/KAP3 under a MARK2 (microtubule affinity-regulating kinases 2) phosphorylation cascade in axonal cargo trafficking (Fréal et al. 2019; Ichinose et al. 2019). TRIM46 has been known to be involved in inflammatory response through ubiquitination. It ubiquitinates DUSP1 (Dual-specificity phosphatase 1), which in turn induces activation of NF-κB and MAPK (Mitogen-activated protein kinases) in colonic inflammation (Li et al. 2020). On the other hand, TRIM46 acts as an oncogene in osteosarcoma by interacting with and ubiquitinating peroxisome proliferator-activated receptor alpha (PPARα), resulting in the activation of the NF-κB signaling pathway (Jiang et al. 2020). Despite the many reports describing the various molecular functions of TRIM46 in mammalian cells, its embryological functions have yet to be determined. We thus investigated expression patterns as well as signaling networks governing the transcription of *trim46a* in zebrafish embryos. We propose a signaling pathway including Sonic Hedgehog (SHH) and Foxa2, which controls transcription of *trim46a*.

## Materials and methods

### Adult zebrafish care and embryos

Wild types of adult zebrafish were maintained at 28.5°C with 10 h dark/14 h light cycles. Embryos were obtained through natural spawning and raised, and staged as described previously (Kimmel et al. 1995; Westerfield 2000). The pigmentation of embryos for whole-mount *in situ* hybridization (WISH) was blocked by treating them with 0.002% phenylthiourea (PTU) after onset of somitogenesis.

### Sequence analysis

The sequences and additional information of zebrafish *trim46a* and other orthologs were obtained by searching the database of National Center for Biotechnology Information (NCBI) (http://www.ncbi.nim.nih.gov/). Amino acid sequences of *trim46a* and other orthologs were aligned as previously described (Choe et al. 2020) to identify the similarity among them.

### RNA isolation, cDNA synthesis and reverse transcription-PCR (RT-PCR)

Total RNA isolation, cDNA synthesis and reverse transcription polymerase chain reaction (RT-PCR) were performed as described in (Kang et al. 2014). The primers were used for PCR to analyze the zebrafish *trim46a* specific template (228 bp) in different stages of embryos, forward primer; 5′-GGACTGCGTTAAATTGGACCTCTCAG-3′ and reverse primer; 5′-ATCGCTGAAACTGTGCCGTTGTTC-3′, and for β-actin (328 bp), as an internal control for this experiment, forward primer; 5′-GCCCATCTATGAGGGTTACG-3′ and reverse primer; 5′-GCAAGATTCCATACCCAGGA-3′. Upon completion of the PCR reaction, every set of reaction was confirmed with running 1% agarose gel in TE buffer using gel electrophoresis. After cloning in pGEM®T-easy vector and confirmation with the digestion process, plasmid construct was sent for sequencing to SolGent Co. Ltd. The protocols were established and applied as previously in our laboratory (Kumar et al. 2017).

### Whole-mount *in situ* hybridization

Whole-mount *in situ* hybridization (WISH) was performed following a standard procedure with minor modifications as described in (Thisse et al. 1993; Jung et al. 2019). After confirming the sequences in the plasmid construct, cloned *trim46a* construct was linearized with restriction enzyme. Then DIG-labeled antisense and sense probe of *trim46a* were synthesized using the DIG RNA Labeling Kit (SP6/T7) (Roche, USA). Embryos were fixed in 4% paraformaldehyde (PFA) overnight, and dehydrated in 100% methanol. All images and their magnification were captured with Leica MZ16 microscopy systems.

### Bioinformatic analysis of putative promoter regions

Promoter region sequences (3899 bp) of *trim46a* were obtained from National Center for Biotechnology Information (NCBI) (http://www.ncbi.nim.nih.gov/) and searched for the occurrences of binding motifs for transcription factors (TF) as defined by Transfac database (Wingender et al. 1996).

### Treatment of cyclopamine to zebrafish embryos

Embryos were staged and synchronized at 4 hpf by discarding any embryos not at sphere stage, and then incubated beginning at 4 hpf until 24 hpf in embryo medium containing 20 μM and 60 μM cyclopamine respectively (LC Laboratories), diluted from a 10 mM stock in ethanol, at 28.5°C. Control embryos were treated with an equivalent concentration of ethanol. Embryos were raised at low density (no more than 50 embryos/100 mm Petri dish) to ensure developmental synchrony across individuals (Kay et al. 2005), and manually dechorionated at 24 hpf. Phenylthiourea (PTU), normally used to block the pigmentation of embryos for WISH, was not treated to avoid interference in cyclopamine treatment. To stop the treatment, embryos at 24 hpf were rinsed three times in fresh embryo medium, and fixed for WISH analysis.

### Quantification of expression level of *foxa2* and *trim46a*

To provide objective and quantitative assessments of *foxa2* and *trim46a* expression, the total area of WISH staining was examined and analyzed by ImageJ software (National Institutes of Health, Bethesda, MD). First, the images were converted to 8-bit grayscale and the area to be measured was outlined, and the sum of intensity (reaction product plus background) was determined in this region of interest (ROI). Next, the outline was moved to an adjacent region of the embryo that showed no reaction product and the background intensity was determined. This was then subtracted from the ROI. Using the sum intensity and dividing by the ROI pixel area, the mean intensity of an embryo was calculated. The average mean intensity of each gene was obtained from 5 to 15 embryos in each group.

### Statistical analysis

Statistically significant differences between the two groups were determined using the unpaired *t* tests. *P* < 0.05 was considered to indicate a statistically significant difference. GraphPad Prism 9 software was used for data analysis.

## Results

### Identification of *trim46a* and structural analysis of Trim46a

**Figure 1.**
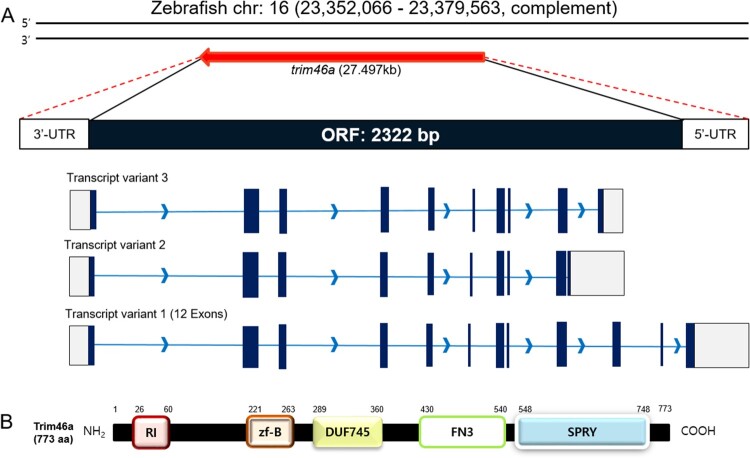
Schematic diagrams of the ORF of *trim46a* and protein structures of Trim46a. (A) Zebrafish *trim46a* is present on chromosome 16 and spans 27,494 bp on the genome. *trim46a* includes 2322 bp of open reading frame (ORF) encoding 773 amino acids long Trim46a. (B) Trim46a contains RING_Ubox superfamily domain (amino acids 26–60), zf-B box domain (amino acids 221–263), DUF745 superfamily domain (amino acids 289–360), FN3 domain (amino acids 430–540), and SPRY superfamily domain (amino acids 548–748).

To identify *trim46a*, an ortholog of *TRIM46* in zebrafish, NCBI database was used as reference. Zebrafish *trim46a* (Gene ID;569905) is mapped to chromosome 16 and located in NC_007127.7 (23,352,066, 23,379,563, complement) which spans about 27.497 kb of nucleotides. *trim46a* transcript consists of 4136 bp containing the open reading frame (ORF) between 340 bp and 2661 bp, which encodes 773 amino acids (Figure 1(A)). Protein structure of Trim46a analyzed by NCBI Conserved Domain Search with the default parameters found that Trim46a consists of the RING_Ubox superfamily domain (105 a. a), zf-B box domain (43 a. a), DUF745 superfamily domain (72 a. a), FN3 domain (111 a. a) and SPRY superfamily domain (201 a. a) (Figure 1(B)). Comparative analysis of Trim46 orthologs among zebrafish, mouse (*M. musculus*), and human (*H. sapiens*) using CLUSTAL OMEGA under default parameters for multiple sequence alignment demonstrated that zebrafish Trim46a shares homology with mouse and human by 58.96% and 59.36%, respectively (data not shown).

### Zygotic transcripts of *trim46a* are restricted to the neural plate, telencephalon, mesencephalon, and hindbrain in embryos at 24 hpf

**Figure 2.**
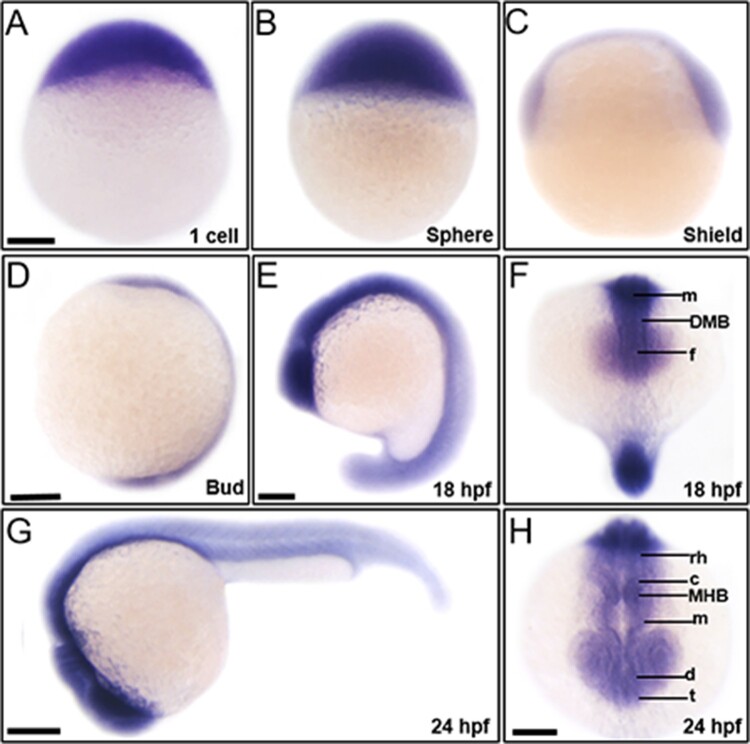
Spatiotemporal expression patterns of *trim46a* in zebrafish embryos at 1 cell, sphere, shield, bud, 18 hpf and 24 hpf. *trim46a* transcripts were distributed in the precursor region of brain along the central nervous system. (A) Maternal messages of *trim46a* were present at 1-cell stage. (B) *trim46a* transcripts were abundant in the deep cell layer (DEL), enveloping layer (EVL) and I-YSL (yolk syncytial layer) at sphere stage. (C) *trim46a* transcripts were localized to the ventral & dorsal region at shield stage. (D) *trim46a* transcripts were abundant in the central nervous system at bud stage. (E and F) *trim46a* transcripts were evenly distributed in the brain region. (G and H) Lateral (G) and anterior (H) view of zebrafish embryos at 24 hpf; *trim46a* messages were restricted to the forebrain through the telencephalon, diencephalon, midbrain, MHB, cerebellum and rhombomere. All embryos were collected synchronously from WT zebrafish for WISH analysis at the corresponding stages. MHB, midbrain-hindbrain boundary; DMB, diencephalic-mesencephalic boundary; c, cerebellum; rh, rhombomere; t, telencephalon; m, midbrain; d, diencephalon; f, forebrain. (A–H) Scale bars: 50 μm.

To elucidate contribution of *trim46a* to vertebrate embryogenesis, we initially examined spatiotemporal expression of *trim46a* in zebrafish embryos at various developmental stages. Analysis of RT-PCR (Reverse Transcription Polymerase Chain Reaction) exhibited that *trim46a* transcripts were abundant at 1-cell stage, and the expression persisted until 24 hpf, indicating that it is maternally and zygotically expressed (data not shown). Spatiotemporal expression patterns of *trim46a* were analyzed with whole-mount *in situ* hybridization (WISH) using *trim46a*-specific digoxigenin-labeled RNA probe in zebrafish embryos at 1-cell (Figure 2(A)), sphere (Figure 2(B)), and shield (Figure 2(C)) stages. *trim46a* transcripts were present as maternal messages while the zygotic transcripts were localized to the central nervous system at bud stage and 18 hpf (Figure 2(D, E)). *trim46a* transcripts were restricted to the neural plate, forebrain, midbrain, and hindbrain at 18 hpf (Figure 2(F)) and further to the eyes, telencephalon, diencephalon, midbrain, MHB (Midbrain-Hindbrain Boundary), cerebellum and rhombomeres (Figure 2(G,H)).

### *Trim46a* promoter contains *cis*-acting elements preferentially binding to Foxa2

**Figure 3.**
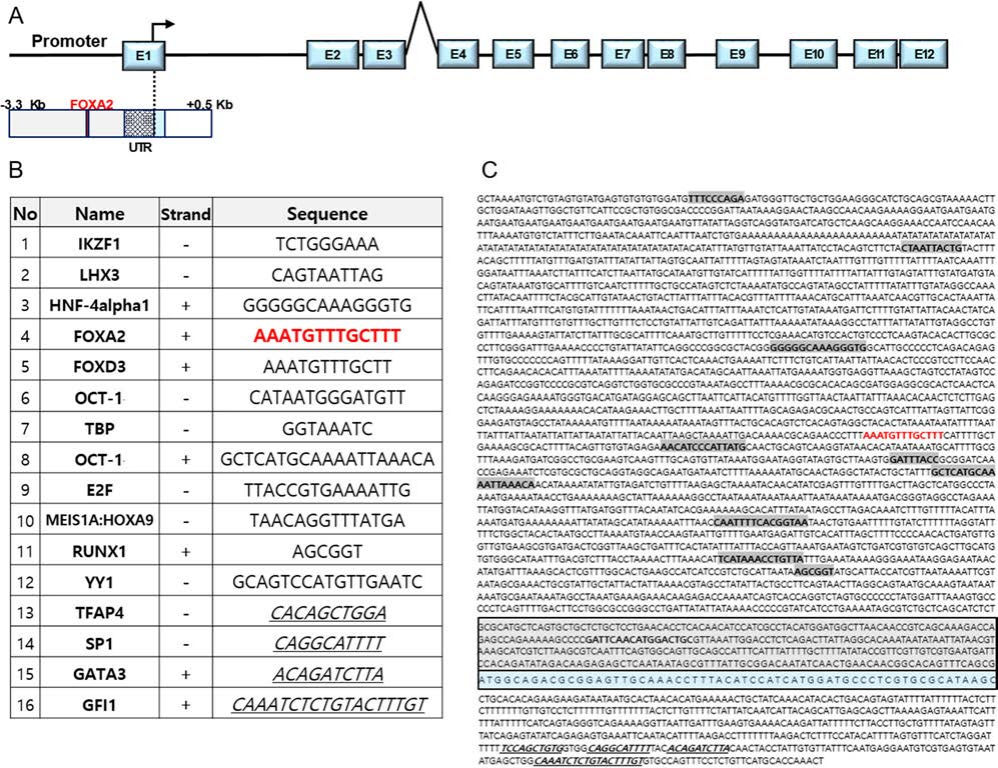
Putative *cis*-acting elements in the promoter region (3899 bp) of *trim46a*. Putative *cis*-acting elements defined by the Transfac database (Wingender et al. 1996). (A) Schematic representation of zebrafish *trim46a* genomic region. Twelve exons (E1 to E12) and eleven introns are depicted; the translation initiation site is indicated with an arrow. (B) List of the sixteen transcription factors which might bind to their corresponding response elements within the *trim46a* promoter (3899 bp). (C) The putative transcription factors are highlighted in bold and blue color represents exon1 (Cut-off *p*-value = 0).

To identify potential regulatory elements of *trim46a* transcription, 5’-upstream (3,000 bp long) region from the transcriptional start site and 500bp of the first intron were analyzed by Transfac database (Wingender et al. 1996). Various *cis*-acting elements were identified; Ikaros family zinc finger protein 1 (IKZF1), LIM homeobox gene 3 (LHX3), Hepatocyte nuclear factor 4 alpha 1 (HNF-4alpha1), Forkhead box A2 (FOXA2), Forkhead box D3 (FOXD3), Octamer transcription factor 1 (OCT-1), TATA-binding protein (TBP), E2F, Meis1A:Hoxa9 and Runt-related transcription factor 1 (RUNX1) (Figure 3(B)). Among the candidate transcriptional factors, Foxa2 was selected as a potential regulator of the transcription of *trim46a* because its DNA recognition domain is well conserved in zebrafish (-1244 bp: AAATGTTTGCTTT), mouse (-1618 bp: AGTCAATAGTGCG) and human (-458 bp: GAGGAAATAATGCG) (data not shown). Forkhead box (FOX) proteins comprise a large family of transcription factors (TFs), members of which display functional diversity and participate in cellular processes ranging from development to immunity and metabolism (Hannenhalli and Kaestner 2009; Lam et al. 2013).

### Inhibition of Sonic Hedgehog (SHH) signaling caused developmental defects in the midbrain at 24 hpf

**Figure 4.**
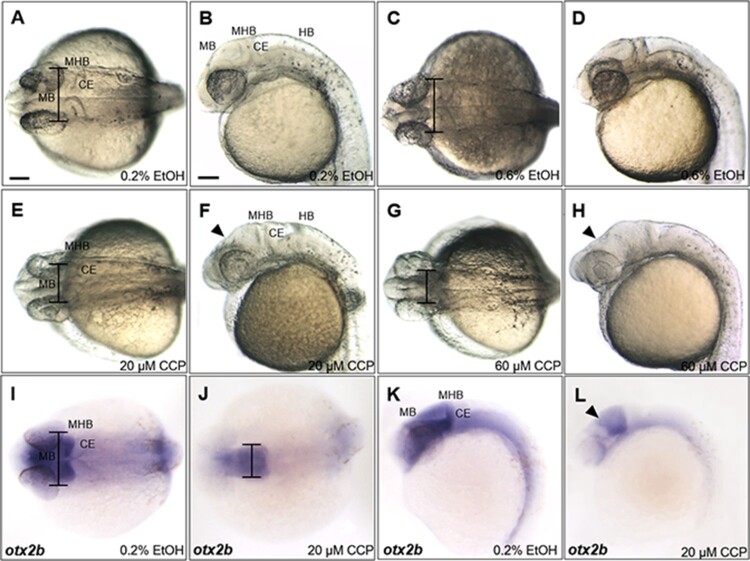
Inhibition of SHH signaling with cyclopamine resulted in developmental defects in the midbrain of zebrafish embryos at 24 hpf. (A–D) Zebrafish embryos at 24 hpf which were treated 0.2% and 0.6% EtOH from 4 hpf. 0.6% EtOH-exposed embryos showed similar phenotypes of midbrain and MHB in comparison to those of 0.2% EtOH-exposed embryos. (E and F) Cyclopamine-treated embryos (20 μM) showed reduction in width of the midbrain and dorsally shrunken midbrain in comparison to those of EtOH controls. (G and H) 60 μM cyclopamine-treated embryos exhibited severe defects in the midbrain. (I–L) Spatiotemporal expression patterns of *otx2b* in the midbrain of cyclopamine-treated embryos at 24 hpf. CCP, cyclopamine. (A–L) Scale bars: 50 μm.

Organizing midbrain signaling centers is known to be tightly associated with SHH signaling in chick embryos (Bayly et al. 2012) while Foxa2 is a direct downstream target of SHH pathway and pharmacologically blocked by cyclopamine (Chen et al. 2002; Schäfer et al. 2007). Because the upstream promoter region of *trim46a* contains a binding site for Foxa2 (Figure 3), it is conceivable that SHH signaling might control transcription of *trim46a* via Foxa2. To test this possibility, an inhibitor of SHH, cyclopamine was treated to zebrafish embryos at 4 hpf through 24 hpf. Embryos treated with cyclopamine at 20 μM and 60 μM demonstrated severe shrinkage in the midbrain along the dorso-ventral axis, accompanying notable reduction in width of the midbrain at 24 hpf (Figure 4(A–H)). Developmental defects of the midbrain in the cyclopamine-treated embryos were further analyzed with a midbrain marker *otx2b* (Li et al. 1994). *otx2b* transcripts detected in the midbrain were outstandingly lower than those in the ethanol-treated controls at 24 hpf (Figure 4(I–L)), suggesting that inhibition of SHH signaling with cyclopamine hinders proper development of the midbrain. Considering the presence of Foxa2 binding site in the upstream promoter region of *trim46a* and SHH as an upstream regulator of Foxa2, it is highly probable that *trim46a* defines development of the midbrain via SHH-Foxa2 signaling pathway in zebrafish embryos.

### Inhibition of SHH reduced the level of transcripts of *foxa2* and *trim46a* in the embryos at 24 hpf

**Figure 5.**
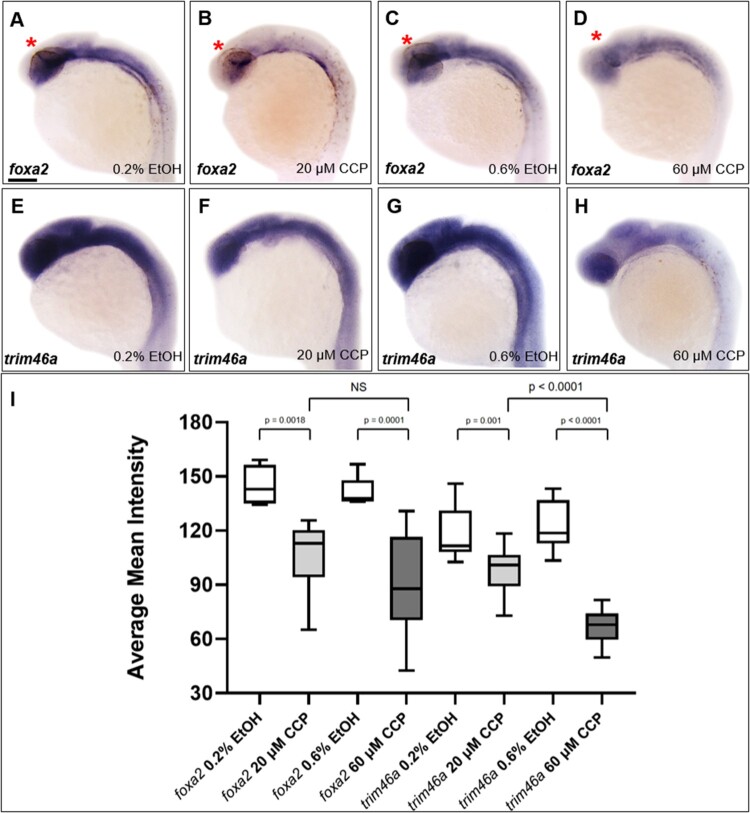
Inhibition of SHH signaling by cyclopamine decreased the level of *trim46a* transcripts in dose-dependent manners. (A–D) WISH analysis using *foxa2* specific probe detected *foxa2* transcripts in the midbrain at 24 hpf. Level of *foxa2* transcripts decreased in the embryos treated with cyclopamine versus the control. (E–H) WISH analysis of cyclopamine-treated embryos using *trim46a* as a probe. Cyclopamine caused reduction in the level of *trim46a* transcripts in comparison to those of EtOH control in dose-dependent manners. (I) Quantification of *foxa2* transcripts in the ZLI, and *trim46a* transcripts in the forebrain, midbrain and cerebellum at 24 hpf using ImageJ software. CCP, cyclopamine. (A–H) Scale bars: 50 μm.

To determine if the inhibition of SHH regulates transcription of *trim46a*, spatiotemporal expression patterns of *trim46a* were analyzed in zebrafish embryos treated with cyclopamine. Spatiotemporal expression patterns of *foxa2* were initially studied to ask if Foxa2, a downstream target of SHH signaling regulates transcription of *trim46a* via the putative Foxa2 binding site in the promoter. Upon the treatment of cyclopamine to zebrafish embryos, level of *foxa2* transcripts was significantly reduced in the ZLI (Figure 5(B,D)) compared to that of the control at 24 hpf (Figure 5(A,C)). In order to examine if the reduced transcripts of *foxa2* caused any changes in transcription *trim46a*, spatiotemporal expression patterns of *trim46a* in the cyclopamine-treated embryos were analyzed with WISH. Level of *trim46a* transcripts were decreased in the hypothalamus, midbrain and MHB in dose-dependent manners (Figure 5(F,H)). Furthermore, staining intensity of the transcripts from *trim46a* and *foxa2* were measured for validation of the anatomical territories in the brain by employing ImageJ (National Institutes of Health, Bethesda, MD) analysis software. Region of interest (ROI) consists of the forebrain, midbrain and cerebellum except for the rhombomeres. Calculation of the mean intensity indicated that inhibition of SHH signaling by cyclopamine reduced level of *trim46a* transcripts more significantly than that of *foxa2* in the ROI of the zebrafish embryos at 24 hpf (Figure 5(I)). Based upon all the observations, it is highly probable that proper expression of *trim46a* via SHH signaling is essential to development of the midbrain in zebrafish embryos.

## Discussion

### SHH signaling is required for *trim46a* to develop the ZLI in midbrain in zebrafish embryogenesis

Cyclopamine led to the repression of genes specifically associated with hedgehog signaling (Incardona et al. 1998; Chen et al. 2002). Formation of the ZLI in midbrain is closely related to temporal adaptation of gradient SHH for the pre-thalamic and thalamic gene expression domains during diencephalic patterning (Krauss et al. 1993; Balaskas et al. 2012). Our data in this report provide evidences that the cyclopamine-treated embryos exhibited dorsally reduced transcripts of *foxa2* in the ZLI (Figure 5(B,D)). This, in turn, implies that acquisition of *trim46a* expression is dependent on direct SHH signaling for proper neurogenesis of the diencephalon, which requires the ZLI-derived SHH signaling (Kiecker and Lumsden 2004).

### Foxa2 is a putative transcriptional regulator of *trim46a* in response to SHH signaling

TRIM46 promoted cell growth and inhibited apoptosis of osteosarcoma cells by activating NF-κB signaling pathway through the ubiquitination of PPAR (Peroxisome proliferator-activated receptor). TRIM46 induces ubiquitination of PPAR-α and regulates cell viability, cell cycle, and apoptosis of OS (Jiang et al. 2020). PPARs regulate many cellular processes in the CNS, for instance, PPARγ regulates neural cell differentiation, death, and inflammation, in addition to playing a neuroprotective role in human neural stem cells (Chiang et al. 2016). Two duplicate paralogs of mammalian PPARA namely, Pparαa and Pparαb are present in the zebrafish genome. Both Pparαa and Pparαb are essential regulators of the proliferation of neuronal and glial precursors in zebrafish embryos (Hsieh et al. 2018). Based on our findings that trim46a is implicated in zebrafish neurogenesis, further study of its relevance to Pparαa and Pparαb would be an interesting topic. In addition, bioinformatics studies in this report discovered a putative Foxa2 binding site in the upstream region of *trim46a* (Figure 3). Furthermore, the repression of Foxa2 by cyclopamine caused the transcriptional repression of *trim46a* (Figure 5), which resulted in the developmental defects of the ZLI of zebrafish embryos (Figure 4). Most paralogous FOX proteins bind to *cis*-acing element 5′-RYAAAYA-3′ (R = A or G, Y = C or T) (Koh et al. 2009; Chen et al. 2016). The conserved C-terminus of FOXA proteins interact with histones H3 and H4 and overcome physical barrier of nucleosomes (Clark et al. 1993). FOXA2, which belongs to pioneer transcription factors (PTFs) has ability to engage their target sequence on nucleosomal DNA and initiate gene expression that confers cell identities (Soufi et al. 2015). Foxa2 is critical to continued survival of the mesodiencephalic dopaminergic neurons (Kittappa et al. 2007) and cooperates with Lmx1a and Lmx1b to coordinate the specification of dopaminergic neurons and control of floor plate cell differentiation in the developing mesencephalon (Nakatani et al. 2010). In particular, it is most likely that Foxa2 contributes to the specification of dopaminergic neurons and the control of floor plate cell differentiation in the developing mesencephalon by regulating the transcription of *trim46a* in the zebrafish embryos.

## References

[CIT0001] Balaskas N, Ribeiro A, Panovska J, Dessaud E, Sasai N, Page KM, Briscoe J, Ribes V. 2012. Gene regulatory logic for reading the Sonic Hedgehog signaling gradient in the vertebrate neural tube. Cell. 148:273–284. 22265416 10.1016/j.cell.2011.10.047 PMC3267043

[CIT0002] Bayly RD, Brown CY, Agarwala S. 2012. A novel role for FOXA2 and SHH in organizing midbrain signaling centers. Dev Biol. 369:32–42. 22750257 10.1016/j.ydbio.2012.06.018 PMC3423079

[CIT0003] Chen X, Ji Z, Webber A, Sharrocks AD. 2016. Genome-wide binding studies reveal DNA binding specificity mechanisms and functional interplay amongst Forkhead transcription factors. Nucleic Acids Res. 44:1566–1578. 26578569 10.1093/nar/gkv1120 PMC4770209

[CIT0004] Chen JK, Taipale J, Cooper MK, Beachy PA. 2002. Inhibition of Hedgehog signaling by direct binding of cyclopamine to smoothened. Genes Dev. 16:2743–2748. 12414725 10.1101/gad.1025302 PMC187469

[CIT0005] Chiang MC, Nicol CJ, Cheng YC, Lin KH, Yen CH, Lin CH. 2016. Rosiglitazone activation of PPARγ-dependent pathways is neuroprotective in human neural stem cells against amyloid-beta-induced mitochondrial dysfunction and oxidative stress. Neurobiol Aging. 40:181–190. 26973118 10.1016/j.neurobiolaging.2016.01.132

[CIT0006] Choe S, Huh TL, Rhee M. 2020. Trim45 is essential to the development of the diencephalon and eye in zebrafish embryos. Anim Cells Syst. 24:99–106. 10.1080/19768354.2020.1751281 PMC7241540 32489689

[CIT0007] Clark KL, Halay ED, Lai E, Burley SK. 1993. Co-crystal structure of the HNF-3/fork head DNA-recognition motif resembles histone H5. Nature. 364:412–420. 8332212 10.1038/364412a0

[CIT0008] Curcio M, Bradke F. 2015. Microtubule organization in the axon: TRIM46 determines the orientation. Neuron. 88:1072–1074. 26687215 10.1016/j.neuron.2015.12.006

[CIT0009] Deshaies RJ, Joazeiro CA. 2009. RING domain E3 ubiquitin ligases. Annu Rev Biochem. 78:399–434. 19489725 10.1146/annurev.biochem.78.101807.093809

[CIT0010] Fréal A, Rai D, Tas RP, Pan X, Katrukha EA, van de Willige D, Stucchi R, Aher A, Yang C, Altelaar AFM, et al. 2019. Feedback-driven assembly of the axon initial segment. Neuron. 104:305–321. 31474508 10.1016/j.neuron.2019.07.029 PMC6839619

[CIT0011] Hannenhalli S, Kaestner KH. 2009. The evolution of Fox genes and their role in development and disease. Nat Rev Genet. 10:233–240. 19274050 10.1038/nrg2523 PMC2733165

[CIT0012] Harterink M, Vocking K, Pan X, Soriano Jerez EM, Slenders L, Fréal A, Tas RP, van de Wetering WJ, Timmer K, Motshagen J, et al. 2019. TRIM46 organizes microtubule fasciculation in the axon initial segment. J Neurosci. 39:4864–4873. 30967428 10.1523/JNEUROSCI.3105-18.2019 PMC6670255

[CIT0013] Hsieh YC, Chiang MC, Huang YC, Yeh TH, Shih HY, Liu HF, Chen HY, Wang CP, Cheng YC. 2018. Pparα deficiency inhibits the proliferation of neuronal and glial precursors in the zebrafish central nervous system. Dev Dyn. 247:1264–1275. 30358936 10.1002/dvdy.24683

[CIT0014] Ichinose S, Ogawa T, Jiang X, Hirokawa N. 2019. The spatiotemporal construction of the axon initial segment via KIF3/KAP3/TRIM46 transport under MARK2 signaling. Cell Rep. 28:2413–2426. 31461655 10.1016/j.celrep.2019.07.093

[CIT0015] Incardona JP, Gaffield W, Kapur RP, Roelink H. 1998. The teratogenic veratrum alkaloid cyclopamine inhibits sonic hedgehog signal transduction. Development. 125:3553–3562. 9716521 10.1242/dev.125.18.3553

[CIT0016] Jiang W, Cai X, Xu T, Liu K, Yang D, Fan L, Li G, Yu X. 2020. Tripartite motif-containing 46 promotes viability and inhibits apoptosis of osteosarcoma cells by activating NF-B signaling through ubiquitination of PPAR. Oncol Res. 28:409–421. 32295675 10.3727/096504020X15868639303417 PMC7851538

[CIT0017] Jung J, Udhaya Kumar S, Choi I, Huh TL, Rhee M. 2019. Znf76 is associated with development of the eyes, midbrain, MHB, and hindbrain in zebrafish embryos. Anim Cells Syst. 23:26–31. 10.1080/19768354.2018.1557744 PMC6394295 30834156

[CIT0018] Kang N, Won M, Rhee M, Ro H. 2014. Siah ubiquitin ligases modulate nodal signaling during zebrafish embryonic development. Mol Cells. 37:389–398. 24823357 10.14348/molcells.2014.0032 PMC4044310

[CIT0019] Kay JN, Link BA, Baier H. 2005. Staggered cell-intrinsic timing of ath5 expression underlies the wave of ganglion cell neurogenesis in the zebrafish retina. Development. 132:2573–2585. 15857917 10.1242/dev.01831

[CIT0020] Kiecker C, Lumsden A. 2004. Hedgehog signaling from the ZLI regulates diencephalic regional identity. Nat Neurosci. 7:1242–1249. 15494730 10.1038/nn1338

[CIT0021] Kimmel CB, Ballard WW, Kimmel SR, Ullmann B, Schilling TF. 1995. Stages of embryonic development of the zebrafish. Dev Dyn. 203:253–310. 8589427 10.1002/aja.1002030302

[CIT0022] Kittappa R, Chang WW, Awatramani RB, McKay RD. 2007. The foxa2 gene controls the birth and spontaneous degeneration of dopamine neurons in old age. PLoS Biol. 5:e325. 18076286 10.1371/journal.pbio.0050325 PMC2121110

[CIT0023] Kleiger G, Mayor T. 2014. Perilous journey: a tour of the ubiquitin-proteasome system. Trends Cell Biol. 24:352–359. 24457024 10.1016/j.tcb.2013.12.003 PMC4037451

[CIT0024] Koh KP, Sundrud MS, Rao A. 2009. Domain requirements and sequence specificity of DNA binding for the forkhead transcription factor FOXP3. PLoS One. 4:e8109. 19956618 10.1371/journal.pone.0008109 PMC2779587

[CIT0025] Krauss S, Concordet JP, Ingham PW. 1993. A functionally conserved homolog of the drosophila segment polarity gene HH is expressed in tissues with polarizing activity in zebrafish embryos. Cell. 75:1431–1444. 8269519 10.1016/0092-8674(93)90628-4

[CIT0026] Kumar A, Huh TL, Choe J, Rhee M. 2017. Rnf152 is essential for NeuroD expression and delta-notch signaling in the zebrafish embryos. Mol Cells. 40:945–953. 29276941 10.14348/molcells.2017.0216 PMC5750713

[CIT0027] Lam EW, Brosens JJ, Gomes AR, Koo CY. 2013. Forkhead box proteins: tuning forks for transcriptional harmony. Nat Rev Cancer. 13:482–495. 23792361 10.1038/nrc3539

[CIT0028] Li Y, Allende ML, Finkelstein R, Weinberg ES. 1994. Expression of two zebrafish orthodenticle-related genes in the embryonic brain. Mech Dev. 48(3):229–244. 7893604 10.1016/0925-4773(94)90062-0

[CIT0029] Li Y, Xu S, Xu Q, Chen Y. 2020. Clostridium difficile toxin B induces colonic inflammation through the TRIM46/DUSP1/MAPKs and NF-κB signalling pathway. Artif Cells Nanomed Biotechnol. 48:452–462. 31918570 10.1080/21691401.2019.1709856

[CIT0030] McNab FW, Rajsbaum R, Stoye JP, O’Garra A. 2011. Tripartite-motif proteins and innate immune regulation. Curr Opin Immunol. 23:46–56. 21131187 10.1016/j.coi.2010.10.021

[CIT0031] Meroni G, Diez-Roux G. 2005. TRIM/RBCC, a novel class of ‘single protein RING finger’ E3 ubiquitin ligases. Bioessays. 27:1147–1157. 16237670 10.1002/bies.20304

[CIT0032] Metzger MB, Hristova VA, Weissman AM. 2012. HECT and RING finger families of E3 ubiquitin ligases at a glance. J Cell Sci. 125:531–537. 22389392 10.1242/jcs.091777 PMC3381717

[CIT0033] Nakatani T, Kumai M, Mizuhara E, Minaki Y, Ono Y. 2010. Lmx1a and Lmx1b cooperate with Foxa2 to coordinate the specification of dopaminergic neurons and control of floor plate cell differentiation in the developing mesencephalon. Dev Biol. 339:101–113. 20035737 10.1016/j.ydbio.2009.12.017

[CIT0034] Nalepa G, Rolfe M, Harper JW. 2006. Drug discovery in the ubiquitin-proteasome system. Nat Rev Drug Discov. 5:596–613. 16816840 10.1038/nrd2056

[CIT0035] Ozato K, Shin DM, Chang TH, Morse HC. 2008. TRIM family proteins and their emerging roles in innate immunity. Nat Rev Immunol. 8:849–860. 18836477 10.1038/nri2413 PMC3433745

[CIT0036] Reymond A, Meroni G, Fantozzi A, Merla G, Cairo S, Luzi L, Riganelli D, Zanaria E, Messali S, Cainarca S, et al. 2001. The tripartite motif family identifies cell compartments. EMBO J. 20:2140–2151. 11331580 10.1093/emboj/20.9.2140 PMC125245

[CIT0037] Schäfer M, Kinzel D, Winkler C. 2007. Discontinuous organization and specification of the lateral floor plate in zebrafish. Dev Biol. 301:117–129. 17045256 10.1016/j.ydbio.2006.09.018

[CIT0038] Short KM, Cox TC. 2006. Subclassification of the RBCC/TRIM superfamily reveals a novel motif necessary for microtubule binding. J Biol Chem. 281:8970–8980. 16434393 10.1074/jbc.M512755200

[CIT0039] Soufi A, Garcia MF, Jaroszewicz A, Osman N, Pellegrini M, Zaret KS. 2015. Pioneer transcription factors target partial DNA motifs on nucleosomes to initiate reprogramming. Cell. 161:555–568. 25892221 10.1016/j.cell.2015.03.017 PMC4409934

[CIT0040] Thisse C, Thisse B, Schilling TF, Postlethwait JH. 1993. Structure of the zebrafish snail1 gene and its expression in wild-type, spadetail and no tail mutant embryos. Development. 119:1203–1215. 8306883 10.1242/dev.119.4.1203

[CIT0041] van Beuningen SFB, Will L, Harterink M, Chazeau A, van Battum EY, Frias CP, Franker MAM, Katrukha EA, Stucchi R, Vocking K, et al. 2015. TRIM46 controls neuronal polarity and axon specification by driving the formation of parallel microtubule arrays. Neuron. 88:1208–1226. 26671463 10.1016/j.neuron.2015.11.012

[CIT0042] Versteeg GA, Rajsbaum R, Sánchez-Aparicio MT, Maestre AM, Valdiviezo J, Shi M, Inn KS, Fernandez-Sesma A, Jung J, García-Sastre A. 2013. The E3-ligase TRIM family of proteins regulates signaling pathways triggered by innate immune pattern-recognition receptors. Immunity. 38:384–398. 23438823 10.1016/j.immuni.2012.11.013 PMC3584420

[CIT0043] Westerfield M. 2000. The zebrafish book: A guide for the laboratory use of zebrafish (*Danio rerio*). Eugene: Univ. of Oregon Press.

[CIT0044] Wingender E, Dietze P, Karas H, Knüppel R. 1996. TRANSFAC: a database on transcription factors and their DNA binding sites. Nucleic Acids Res. 24:238–241. 8594589 10.1093/nar/24.1.238 PMC145586

